# Evidence for ethnicity and location as regulators of the newborn blood metabolome: a monozygous twin study

**DOI:** 10.3389/fnut.2023.1259777

**Published:** 2024-01-04

**Authors:** Huimin Jiang, Ting-Li Han, Jing Yang, Yang Yang, Fengdi Wang, Yuelu Chen, Nana Huang, Toby Mansell, Jeffrey M. Craig, Katrina J. Scurrah, Boris Novakovic, Philip N. Baker, Hua Zhang, Yuan Wei, Lianlian Wang, Richard Saffery

**Affiliations:** ^1^Department of Reproductive Medicine Center, The First Affiliated Hospital of Chongqing Medical University, Chongqing, China; ^2^Department of Obstetrics and Gynecology, The Second Affiliated Hospital of Chongqing Medical University, Chongqing, China; ^3^Department of Obstetrics and Gynecology, Peking University Third Hospital, Beijing, China; ^4^Department of Obstetrics and Gynecology, The First Affiliated Hospital of Chongqing Medical University, Chongqing, China; ^5^Chongqing Key Laboratory of Maternal and Fetal Medicine, Chongqing Medical University, Chongqing, China; ^6^Mass Spectrometry Centre of Maternal-Fetal Medicine, Life Science Institution, Chongqing Medical University, Chongqing, China; ^7^Murdoch Children’s Research Institute, and Department of Paediatrics, University of Melbourne, Parkville, VIC, Australia; ^8^The Institute for Mental and Physical Health and Clinical Translation (IMPACT), School of Medicine, Deakin University, Melbourne, VIC, Australia; ^9^Twins Research Australia and Centre for Mental Health, Melbourne School of Population and Global Health, The University of Melbourne, Melbourne, VIC, Australia; ^10^College of Life Sciences, University of Leicester, Leicester, United Kingdom

**Keywords:** monochorionic diamniotic (MCDA) twins, umbilical cord blood plasma, metabolomics, shared and non-shared environmental factors, uncomplicated MCDA twins

## Abstract

**Introduction:**

Monochorionic, diamniotic (MCDA) monozygotic twins share nearly all genetic variation and a common placenta *in utero*. Despite this, MCDA twins are often discordant for a range of common phenotypes, including early growth and birth weight. As such, MCDA twins represent a unique model to explore variation in early growth attributable primarily to *in utero* environmental factors.

**Methods:**

MCDA twins with a range of within-pair birth weight discordance were sampled from the peri/postnatal epigenetic twin study (PETS, Melbourne; *n* = 26 pairs), Beijing twin study (BTS, Beijing; *n* = 25), and the Chongqing longitudinal twin study (LoTiS, Chongqing; *n* = 22). All PETS participants were of European-Australian ancestry, while all Chinese participants had Han ancestry. The average of the birth weight difference between the larger and smaller co-twins for all twin pairs was determined and metabolomic profiles of amino acids, TCA cycle intermediates, fatty acids, organic acids, and their derivatives generated from cord blood plasma by gas chromatograph mass spectrometry. Within and between co-twin pair analyses were performed to identify metabolites specifically associated with discordance in birth weight. Multivariable regression and pathway enrichment analyses between different regions were performed to evaluate the geographical effects on the metabolism of MCDA twin pairs.

**Results:**

PETS twins showed a markedly different metabolic profile at birth compared to the two Chinese samples. Within-pair analysis revealed an association of glutathione, creatinine, and levulinic acid with birth weight discordance. Caffeine, phenylalanine, and several saturated fatty acid levels were uniquely elevated in PETS twins and were associated with maternal BMI and average within pair birth weight, in addition to birth weight discordance. LoTiS twins had higher levels of glutathione, tyrosine, and gamma-linolenic acid relative to PETS and BTS twins, potentially associated with eating habits.

**Conclusion:**

This study highlights the potential role of underlying genetic variation (shared by MZ twins)*, in utero* (non-shared by MZ twins) and location-specific (shared by MZ twins) environmental factors, in regulating the cord blood metabolome of uncomplicated MCDA twins. Future research is needed to unravel these complex relationships that may play a key role in phenotypic metabolic alterations of twins independent of genetic diversity.

## 1 Introduction

The developmental origins of health and disease (DOHaD) hypothesis proposes that differences *in utero* or early life, particularly nutritional imbalance, may lead to permanent changes in physiology, metabolism, and development (1). However, given the complex aetiology of most phenotypes in humans, the ability to disentangle the relative contribution of genetic and environmental factors is problematic. The application of twin study designs represents a unique opportunity to address this issue, particularly as monochorionic diamniotic (MCDA) twins, accounting for 20%–30% of twin pregnancies, are generally considered to be genetically identical with similar mitochondrial DNA copy number and telomere length (2). Despite this, MCDA twins often show different fetal growth and development (3–6). Any phenotypic discordance within MCDA pairs (including at birth) is therefore likely the result of non-shared environmental exposures, independent of genetic variability. MCDA twins provide a unique model for studying the relationship between non-shared environmental factors, and a range of discordant phenotypes, including many diseases (4).

Although there are many twin studies and registries internationally, longitudinal twin cohorts commencing in pregnancy are relatively uncommon, particularly with in-depth biological sampling. The Peri/postnatal Epigenetic Twin Study (PETS) is such a cohort, with 250 pairs of Australian twins and their mothers, established specifically to explore the role of epigenetics in early life via a combination of within and between twin study designs (7, 8). PETS was amongst the first to show discordance in the epigenetic profile (DNA methylation) of MZ twins at birth, arising due to non-shared environmental differences *in utero*. PETS also revealed the heritability of DNA methylation profile to be around 20% using a comparison between MZ and DZ pairs (9, 10). The Beijing twin cohort study (BTS) has enrolled 142 MCDA twin pregnancies to date and previously published a study on the hair metabolite profiles of MCDA twins with sIUGR (11). Yang et al. (11) found that the hair metabolite 2-aminobutyric acid, cysteine, and tyrosine were associated with growth retardation and antioxidant capability. They suggested that the neonatal hair metabolites reflect the intrauterine condition and could be used to evaluate the severity of intrauterine ischemia and hypoxia for sIUGR twins. Recently, this study also reported that the selective fetal growth restriction within twins was characterized by a reduced abundance of *Enterococcus, Acinetobacter*, cysteine, and methionine levels in newborn fecal samples (12). Finally, the Chongqing longitudinal twin study (LoTiS) has recruited over 300 pairs of twins from pregnancy, with follow-up to 3 years of age (13, 14). To date the LoTiS team have published several studies, including a metabolomic study on MCDA twins with selective intrauterine growth restriction (sIUGR) in cord plasma and placental samples (15). Wang et al. (15) found that abnormal amino acid, fatty acid metabolism, and exposure to environmental xenobiotics were related to sIUGR. As anticipated, they concluded that *in utero* growth discordance of MCDA twins is caused by environmental rather than genetic factors. Such studies are relatively rare, and the relevance of findings across different geographical and ethnic settings remains unclear.

Metabolomics examines the collection of small molecular compounds involved in the metabolism of organisms that involves maintaining organisms’ normal function and growth. Small differences in gene sequence or expression as a result of environmental influence are often amplified at the metabolite level. The analysis of metabolites can therefore reveal valuable information on biochemical pathways and mechanisms associated with specific phenotypes (16–18). In relation to MCDA twins at birth, this has the potential to identify intrauterine biochemical processes subject to environmental influence in pregnancy.

This international collaborative study aimed to investigate the metabolic changes of umbilical cord in uncomplicated MCDA twin pairs associated with discordance in birth weight (within-pairs), with a secondary aim to explore the influence of ethnicity and geographic location on metabolomic profile (between-pair).

## 2 Materials and methods

### 2.1 Study participants

We chose three groups of healthy genetically identical diamniotic twins without pregnancy complication (e.g., GDM, PE, or sIUGR) from uneventful pregnancies for this study (summarised in Table 1), which varied according to ethnicity and/or geographical location. This included 26 MCDA twin pregnancies from the Peri/postnatal Epigenetic Twin Study (PETS), recruited from three Melbourne hospitals between January 2007 to September 2009 as previously described (19). A total of 22 MCDA twin pregnancies were similarly recruited from The First Affiliated Hospital of Chongqing Medical University between June 2017 to June 2019 as part of the LoTiS (14). The final group of 25 MCDA twin pregnancies was recruited from the Peking University Third Hospital between September 2017 and December 2018 as part of BTS. Women pregnant with twins were recruited for research at the gestational age of 12–16 weeks and followed by a routine ultrasound examination every 2 weeks post-recruitment. All PETS participants were of European-Australian ancestry, while Chinese participants were all of Han ancestry. Genetic data were not available for either Chinese twin cohort study. The chorionicity of the twin pregnancies was determined via ultrasound or confirmed by experienced obstetricians during delivery. The delivery mode for all participants was cesarean section. MCDA twins with a discordance in fetal birth weight of greater than 20% were excluded from this study. Umbilical cord blood was collected and processed into plasma for all twins within 2 h after delivery (11, 15). Data on conception method for LoTiS and BTS cohorts was obtained from medical records, while this was collected by questionnaire in PETS. Since only IVF and natural conception information were available across all three cohorts, we focused on these two variables. This study was carried out in accordance with the principles set out in the Declaration of Helsinki and approved by the Ethical Committee of Chongqing Medical University (201530), and the Ethical Committee of Peking University Third Hospital. PETS was carried out with appropriate human ethics approvals from the Royal Women’s Hospital (06/21), Mercy Hospital for Women (R06/30), and Monash Medical Centre (06117C), Melbourne. Informed consent was obtained from each participant.

**Table 1:** Comparison of clinical characteristics among Melbourne, Beijing, and Chongqing twin groups.

Characteristics and pregnancy outcomes	PETS	BTS	LoTiS	*p*-value (PETS vs. BTS)	*p*-value (PETS vs. LoTiS)	*p*-value (LoTiS vs. BTS)
Maternal age (years)^a^	32.5 ± 5.0	31.8 ± 3.9	29.6 ± 3.7	0.601	0.031	0.058
Gestational age at delivery (wks)^b^	36 (34, 37)	36 (35, 37)	36.5 (35, 37)	0.969	0.459	0.478
Body mass index (kg/m^2^)^b^	25.25 (22.3, 27.6)	21.7 (19.5, 25.3)	21.075 (18.2, 23.0)	0.027	0.001	0.332
Neonatal sex^c^				0.123	0.845	0.326
Male	10 (38.5%)	16 (64%)	10 (45.5%)			
Female	16 (61.5%)	9 (36%)	12 (54.5%)			
IVF-ET/natural conception^c^				0.673	0.827	0.333
IVF-ET	4 (15.4%)	6 (24.0%)	2 (9.1%)			
Natural conception	22 (84.6%)	19 (76.0%)	20 (90.9%)			
Ethnicity^c^				<0.001	<0.001	1
European-Australian ancestry	26 (100%)	0 (0%)	0 (0%)			
Chinese Han	0 (0%)	25 (100%)	22 (100%)			
Delivery^c^				1	1	1
Cesarean	26 (100%)	25 (100%)	22 (100%)			
Vaginal	0 (0%)	0 (0%)	0 (0%)			
Average birth weight (g)^b^	2428.75 (2112.5, 2747.5)	2,480 (2180.0, 2695.0)	2,560 (2376.3, 2848.8)	0.985	0.159	0.107
Birth weight discordance of twin (g)^b^	250 (144.5, 444.8)	200 (110.0, 280.0)	165 (65.0, 227.5)	0.12	0.015	0.249

a Student’s *t*-test.

b Mann–Whitney *U* test (median).

c Chi-square test; IVF-ET, *in vitro* fertilization & embryo transfer. Data are presented as mean ± standard deviation, median (25th percentile, 75th percentile), or *n* (%).

**Figure 1:**
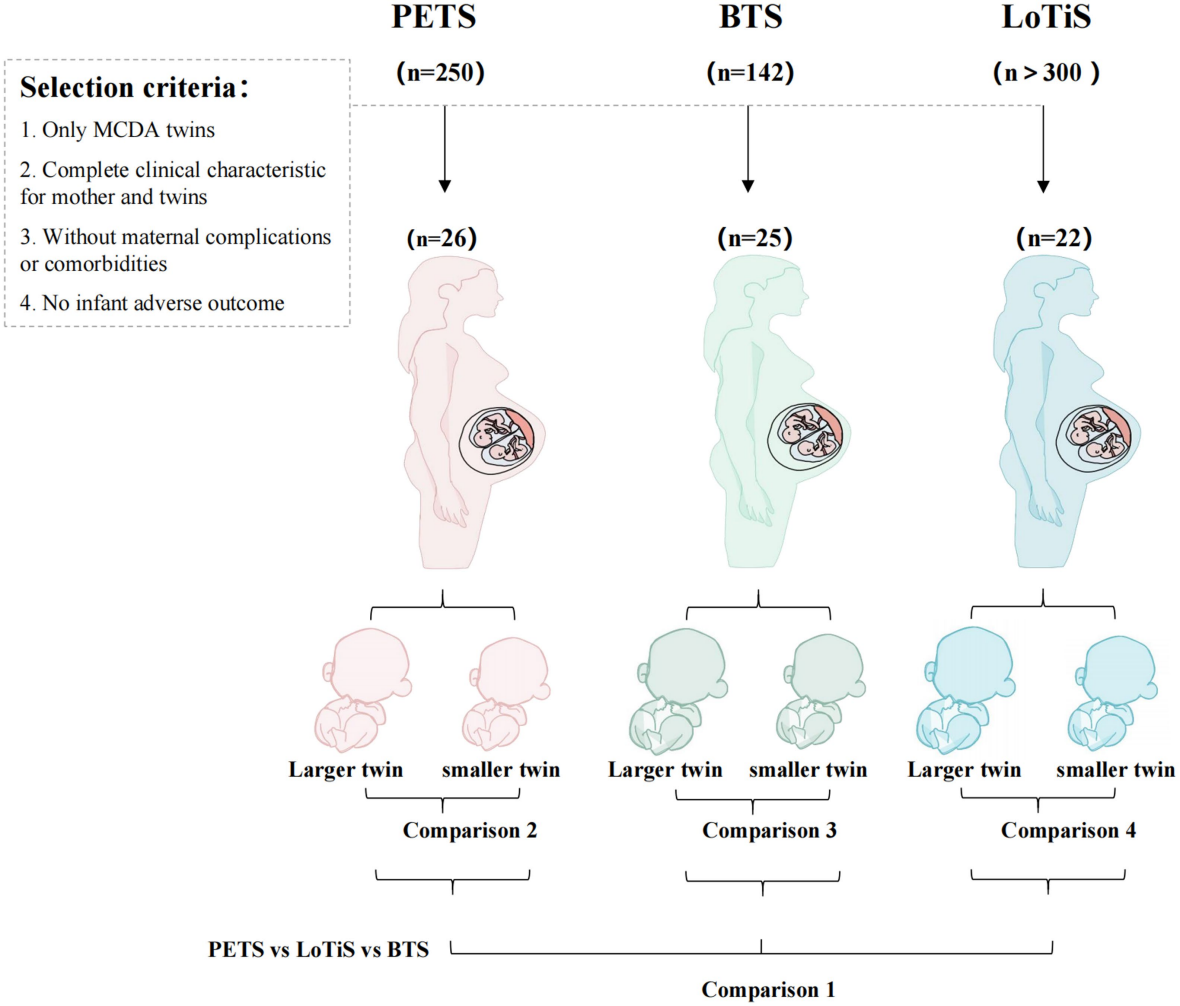
In total, 692 women pregnant with twins were recruited into three independent longitudinal twin cohorts. Of these, 73 pairs of MCDA twin met the selection criteria in the current study. This included 26 PETS, 25 BTS and 22 LoTiS twin pairs, respectively. Comparison 1 compares the cord blood metabolite profiles of PETS, BTS and LoTiS twin groups as a whole. Comparisons 2–4 discriminated metabolites differing between the larger and the smaller twins in each of PETS, BTS and LoTiS groups, respectively.

In total, four different comparisons were undertaken to identify metabolites associated with specific outcomes or exposures (summarized in Figure 1), including location-specific metabolite measures (comparison 1) and within twin pair investigation of birth weight discordance (comparisons 2–4).

### 2.2 Sample collection

For LoTiS, 4 mL of blood was collected from each of the two umbilical veins into EDTA-coated blood collection tubes. These were centrifuged twice at 3,000 rpm for 10 min at 4°C. For BTS, 5 mL of blood was collected from each of the two umbilical veins into EDTA-coated blood collection tubes. These were then centrifuged twice at 1,650 rpm for 10 min at 4°C. For PETS, up to 20 mL of venous umbilical cord blood was collected into EDTA-coated tubes and processed by centrifugation at 1,500 rpm for 10 min. In all studies, blood processing was done within 2–3 h of collection and plasma aliquots were stored at −80°C.

### 2.3 Metabolite extraction of the cord plasma

0.3 μmol of 2,3,3,3-d4-alanine (internal standard) was added to each plasma sample (150 μL). Subsequently, 510 μL of pre-chilled methanol was added and frozen at −20°C for 30 min to facilitate protein precipitation. Plasma supernatants were then centrifuged at 12,000 rpm for 15 min at 4°C and dehydrated in a SpeedVac (Labconco, United States) at room temperature for 7 h, followed by storage at −80°C until chemical derivatization.

### 2.4 Methyl chloroformate derivatization and gas chromatography-mass spectrometry analysis

The extracted samples were chemically derivatized via the methyl chloroformate (MCF) derivatization method, in accordance with the protocol published by Smart et al. (20). MCF derivatized compounds were examined by an Agilent GC-7890B system coupled to a MSD-5977A mass selective detector with the electron impact voltage set at 70 eV. The GC column used for metabolite separation was the ZB-1701 GC capillary column (30 m × 250 μm id × 0.15 μm with 5 m guard column, Phenomenex). The GC temperature was programmed according to the recommendations of Han et al. (21).

### 2.5 GC-MS data mining and data normalization

Metabolomics data were generated in the same lab but at different times. To minimize any impact of batch effects, all samples were prepared using an identical protocol and mass spectrometry approach that included common standards and data normalization approach. Deconvoluting overlapped chromatograms were achieved via the automated mass spectral deconvolution and identification system (AMDIS) (22). Metabolite identifications were then conducted using the in-house MCF mass spectral library established by Silas Villas-Boas’s metabolomics laboratory (20). Specific metabolites were identified based on both their mass spectra matching the mass spectral library and correct chromatographic retention time. The relative concentration of identified metabolites was extracted using MassOmics R package (23) that selected the peak height of a chosen reference ion within a correct retention time window. To minimize batch effects associated with the three independent cohorts, metabolomics data from all samples were normalized together. This was done by normalizing independent metabolite levels to the relative level of the internal standard (2,3,3,3-d4-alanine) in the corresponding sample. The differential dilution of plasma metabolites was corrected by the sum of all ion intensities in a spectrum (TIC). Batch variation was minimized using a sample central median approach by which the median of normalized samples was first determined for each batch independently, then each of the 3 batch medians was aligned into a fixed value, and the distribution of metabolite levels was adjusted accordingly.

### 2.6 Statistical analyses

#### 2.6.1 Baseline statistics

To investigate prenatal clinical characteristics among the pregnant subjects from Chongqing, Beijing, and Melbourne, student’s *t*-test was used to compare normally distributed data, including maternal age. The non-parametric Mann–Whitney *U* test was used to compare non-normally distributed data, which consisted of gestational age at delivery, maternal body mass index (BMI), birth weight discordance, and birth weight. A chi-square test was applied for categorical variables such as neonatal sex and method of conception.

#### 2.6.2 Metabolomics analysis

Metabolomics analyses were calculated using individual twin metabolite profiles. Logistic regression, principal component analysis (PCA), and partial least squares discriminant analysis (PLS-DA) with leave-one-out cross-validation (LOOCV) were performed to compare the umbilical cord plasma metabolome profiles among the three groups of twins as well as for within-pair comparisons in each group using Metaboanalyst 3.0 package for R (24). Mathematical models such as logistic regression (LR), random forest (RF), decision tree (DT), artificial neural network (ANN), support vector machine (SVM), naive bayes (NB) and K-nearest neighbors (KNN) were used to make decisions and classify within larger and smaller co-twin pairs (comparison 2–4). A Venn diagram was plotted using OmicShare tools.^1^ In addition, logistic regression was employed to model differences among Melbourne twins, Beijing twins, and Chongqing twins using the general linear model (glm) in R to identify significant metabolites (*p*-value cut off ≤0.05 and *q*-value cut off ≤0.05, comparison 1). The logistic regression models were also adjusted to account for potential confounding factors BMI and maternal age. Generalized estimating equation (GEE) modeling was implemented to identify umbilical cord plasma metabolites correlated with birth weight discordances within twin pairs, while the correlation between twin pairs using the average birth weight of the twins. Metabolic pathways linked to specific metabolites of interest were reconstructed based on the MetScape addon through Cytoscape (Version 3.9.1). The graphic illustrations of heatmaps, line graphs, and chord plots were created using ggplot2 and GOplot R-packages (25).

## 3 Results

### 3.1 Twin cohort characteristics

Clinical characteristics of the study population are shown in Table 1. There were no significant demographic differences observed between LoTiS and BTS twin groups. Maternal age, gestational age at delivery, neonatal sex, IVF-ET/natural conception, average birth weight and birth weight discordance of twins were similar between PETS and BTS, while maternal BMI was significantly higher in the PETS pregnancies relative to BTS (*p* = 0.027) and LoTiS (*p* = 0.001). Maternal age and birth weight discordance of twin pairs were also significantly higher in PETS relative to LoTiS (*p* = 0.031, and *p* = 0.015 respectively). Despite the neonatal gender having a bias towards one sex in PETS and BTS twin cohort, no obvious difference in neonatal sex was observed when analyzing cord blood metabolome as showed in Supplementary Figures S1A–C.

### 3.2 Metabolomic differences in umbilical cord blood between large and small fetuses from the same region

**Figure 2:**
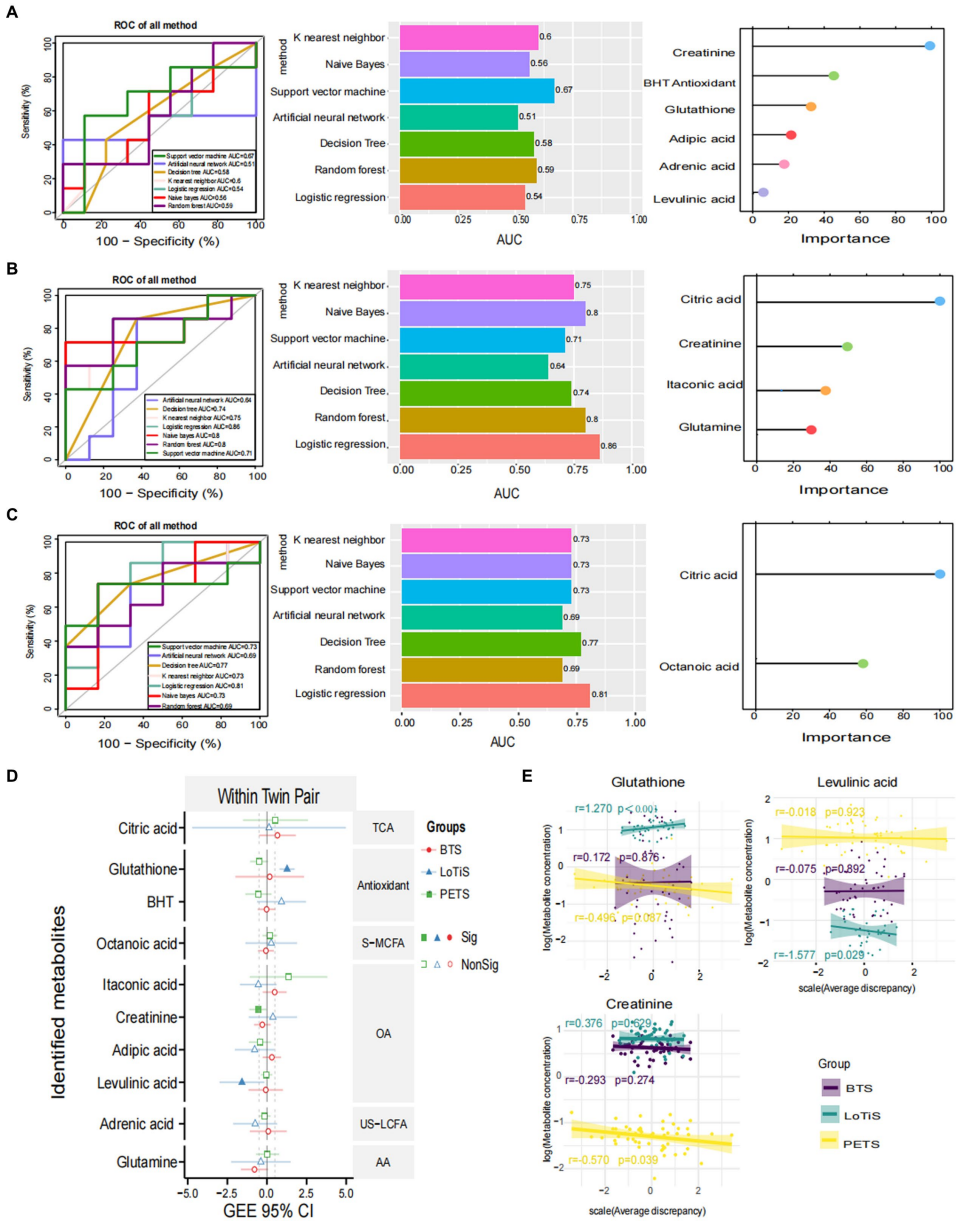
Significant metabolites were nominated and ROC analysis by seven machine learning models of KNN, NB, SVM, ANN, DT, RF, and LR for comparison 2 **(A)**, comparison 3 **(B)**, and comparison 4 **(C)**. ROC curve analysis and corresponding AUC (median with 95% confidence interval) of every model on umbilical cord plasma. The left graphics display the metabolites ranking based on the feature importance of best classified algorithms for each comparison (comparison 2 = SVM: comparison 3 = LR; and comparison 4 = LR). **(D)** The correlation of average birth weight discordance within twin pairs of umbilical cord plasma metabolites detected among Beijing, Chongqing, and Melbourne groups, analyzed using a generalized estimating equation (GEE). The red, blue, and green lines represent the 95% confidence intervals (CI) for Beijing, Chongqing, and Melbourne groups, respectively. The center dotted line in each column indicates 0 correlation; metabolites to the right of the dotted line are positively correlated with birth weight discordance, whereas metabolites to the left of the dotted line are negatively correlated with birth weight discordance. The solid patterns indicate significant correlations, while hollow patterns mean non-significant correlations with *p*-values less than 0.05. **(E)** The correlation between birth weight discordance and significant metabolite concentrations within twin pairs in Beijing (purple lines), Chongqing (green lines), and Melbourne (yellow lines). The shade around the linear regression trendline displays the 95% CI. L, larger twin; S, smaller twin; ROC, receiver operating characteristic; AUC, area under the ROC curve; KNN, K nearest neighbor; NB, naïve bayes; SVM, support vector machine; ANN, artificial neural network; DT, decision tree; RF, random forest; LR, logistics regression; TCA, TCA cycle intermediates; S-MCFA, medium-chain saturated fatty acids; AA, amino acids and amino acid derivatives; US-LCFA, long-chain unsaturated fatty acids; OA, organic acids.

Several approaches were employed to identify metabolites associated with birth weight discordance within pairs. Firstly, partial least squares discriminant analysis (PLS-DA) of the metabolite profile of large vs. smaller twins was carried out within each study. In all three cohorts, a substantial overlap was observed between large and small fetuses. This revealed relatively minor differences between the large and small twins as groups in all three cohorts (Supplementary Figure S2). We also applied seven different machine-learning approaches to shortlist metabolites in order to classify the larger and smaller co-twins within a pair (Figure 2). SVM computed the best area under the ROC curve (AUC) of 0.67 for discriminating PETS co-twins (comparison 2) based on creatinine, BHT, glutathione, adipic acid, adrenic acid and levulinic acid levels. In contrast, LR produced the best AUC of 0.86 for discrimination of larger vs. smaller co-twins in BTS (comparison 3) based on citric acid, creatinine, itaconic acid and glutamine. Similarly, LR generated the best AUC of 0.81 for the discrimination of larger vs. smaller co-twins in LoTiS (comparison 4) using citric acid and octanoic acid. Among the total of 10 identified metabolites that discriminated larger from smaller co-twin by machine-learning approaches (Figures 2A–C), three metabolites exhibited significant correlations with birth weight discordance within twin pairs, as calculated by GEE regression analysis. Particularly, glutathione showed a positive correlation with birth weight discordance in the LoTiS (*r* = 1.270, *p* < 0.001), whereas levulinic acid and creatinine showed negative correlations with birth weight discordance in the LoTiS (*r* = −1.577, *p* = 0.029) and PETS (*r* = −0.570, *p* = 0.039) respectively (Figures 2D,E). However, no common metabolite significantly correlated with birth weight discordance across all three cohorts.

### 3.3 Newborn twins from different locations show a distinct cord blood metabolomic profile

**Figure 3:**
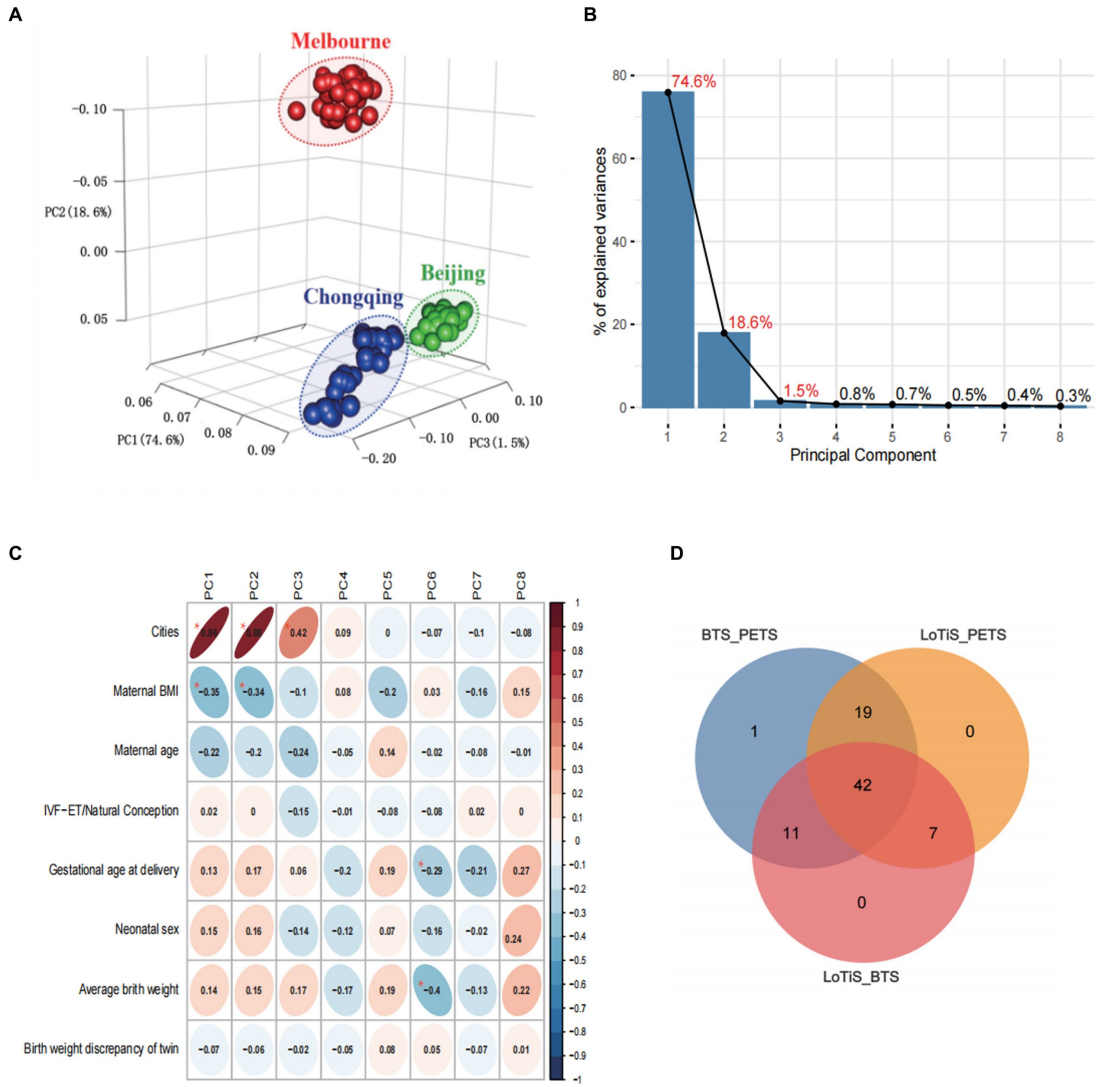
**(A)** Principal component analysis (PCA) of umbilical cord metabolite profiles for cord blood plasma from PETS (Melbourne; red), BTS (Beijing; green) and LoTiS (Chongqing; blue) twins. **(B)** Scree plot showing the relative contribution of PC1-8 to variation within the metabolomic dataset. **(C)** PCA trait correlation shows the degree of correlation of known traits to variation within PCs 1–8, with associated *p*-value (^*^p < 0.001). Shading denotes the direction and strength of correlation. **(D)** Venn diagram of differential metabolites (*p* < 0.05).

Using principal component analysis (PCA), we observed a distinct metabolomic profile for each of the three twin groups in this study (Figure 3A), which was separated clearly by PC1, PC2, and PC3 (accounting for 74.6%, 18.6%, and 1.5% variation within the total dataset respectively; Figure 3B). The variation captured by PC1 and PC2 was largely attributable to differences between PETS twins relative to the two Chinese twin groups (LoTiS and BTS), although PC3 was also associated with study location (Figure 3C). A negative correlation was found between maternal BMI and both PC1 and PC2, while gestational age at delivery and average birth weight showed a negative correlation in PC5 (Figure 3C). No significant associations or correlations of the estimated principal components were observed with maternal age, mode of conception, gestational age at delivery, infant sex, birth weight, or birth weight discordance of the cohorts combined (Figure 3C). Furthermore, the unique or shared metabolites among the three regions were visualized using a Venn diagram (Figure 3D). A total of 42 metabolites showed statistical significance across all regions. Nineteen, eleven, and seven metabolites exhibited shared significance in the PETS-BTS & PETS-LoTiS, BTS-PETS & BTS-LoTiS, and LoTiS-PETS & LoTiS-BETS comparisons, respectively. Only one discriminating metabolite was unique to the comparison between BTS and LoTiS twins.

**Figure 4:**
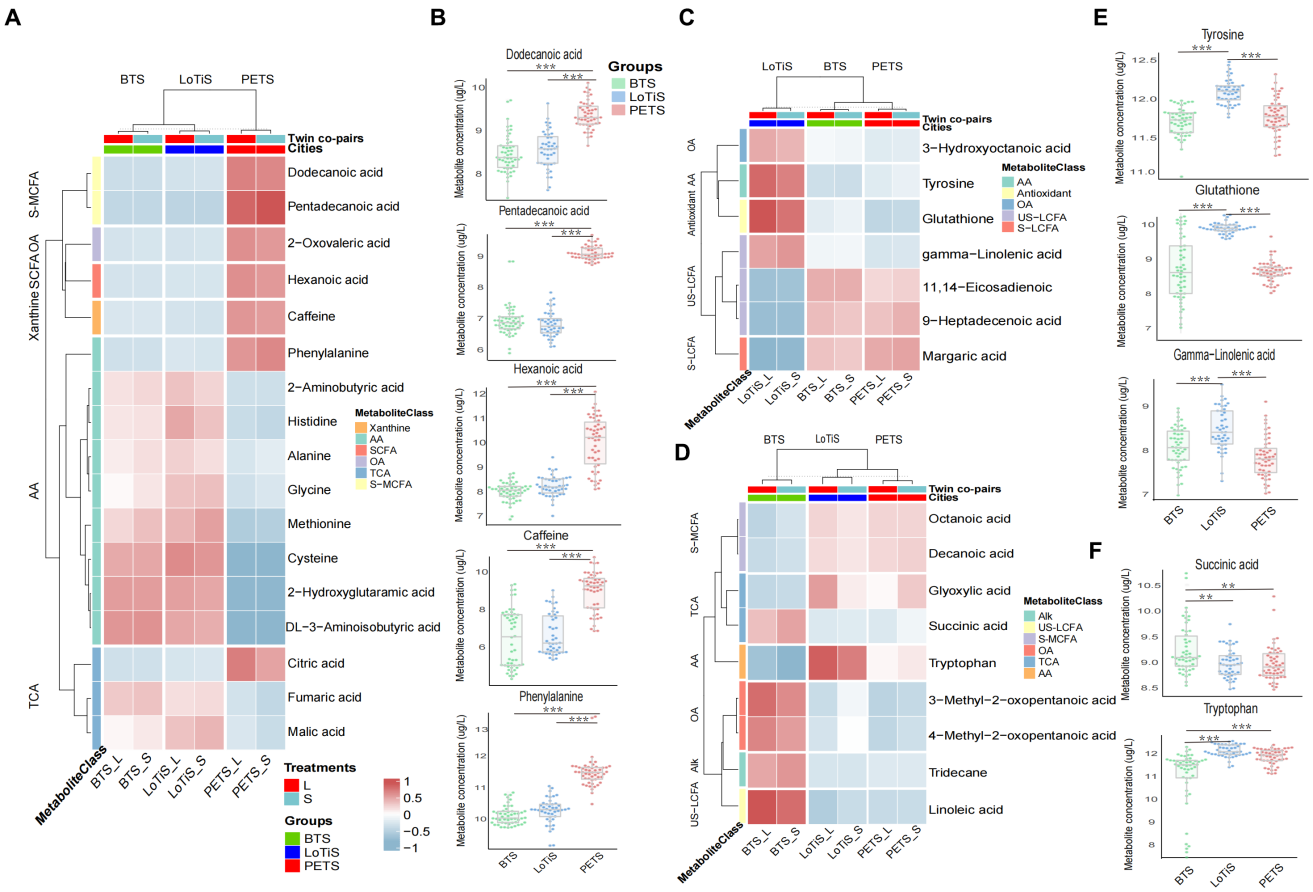
Identification of metabolites specifically different between MCDA twins according to cohort. **(A)** PETS vs. BTS + LoTiS, **(C)** LoTiS vs. BTS + PETS, and **(D)** BTS vs. LoTiS + PETS. The relative concentrations of each relevant metabolite are indicated by colours on a log_2_ scale. Red color corresponds to higher metabolite levels relative to other cohorts, while blue color blocks represent lower metabolite levels. **(B,E,F)** Boxplots of specific metabolites are highlighted in **A,C,D** for each cohort separately. Statistical significance between groups was determined using logistic regression (^*^p < 0.05, ^**^p < 0.01, and ^***^p < 0.001). AA, amino acids and amino acid derivatives; US-MCFA, medium-chain saturated fatty acids; OA, organic acids; SCFA, short-chain saturated fatty acids; TCA, TCA cycle intermediates; S-LCFA, long-chain saturated fatty acids; US-LCFA, long-chain unsaturated fatty acids; Alk, alkanes; S-MCFA, medium-chain saturated fatty acids; L, larger twin; S, smaller twin.

We next identified specific metabolites associated with each cohort and plotted a heatmap of those with a *p*-value <0.05 using logistic regression for the difference between groups. All amino acids and amino acid derivatives (except phenylalanine) and TCA cycle intermediates (except citric acid) were found to be at higher levels in twins of Chinese ethnicity (BTS and LoTiS) relative to the largely European PETS twins, while xanthine (caffeine), fatty acids, phenylalanine and citric acid were significantly lower in BTS and LoTiS relative to PETS (Figure 4A).

In contrast, margaric acid, 11,14-eicosadienoic and 9-heptadecenoic acid showed significantly specifically lower levels in LoTiS (*p*-value less than 0.05 in LoTiS vs. BTS or PETS) (Figure 4C; Supplementary Figure S3), and glutathione, tyrosine, gamma-Linolenic acid, 3-hydroxyoc-tanoic acid were higher (Figures 4C,E). Finally, BTS-specific metabolites, with *p*-values less than 0.05 (BTS vs. LoTiS or PETS), included linoleic acid, 3-methyl-2-oxopentanoic acid, 4-methyl-2-oxopentanoic acid, tridecane, and succinic acid that were higher, while octanoic acid, decanoic acid, glyoxylic acid and tryptophan were lower in BTS twins (Figures 4D,F).

### 3.4 Metabolites associated with average birth weight in three twin cohorts

**Figure 5:**
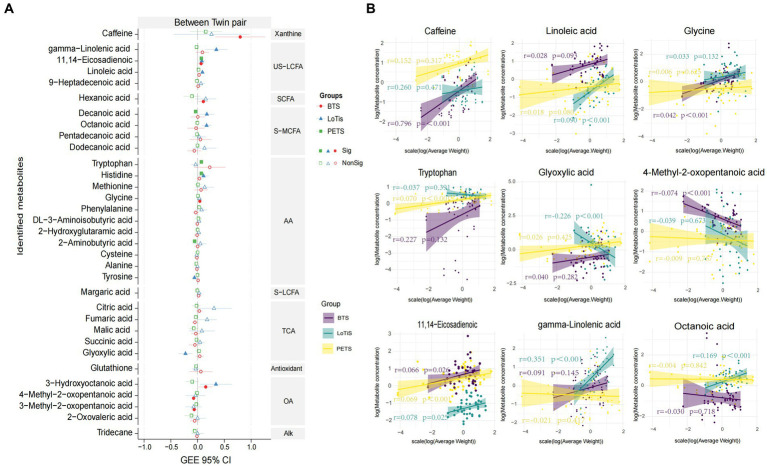
**(A)** Forrest plot showing the correlation of specific metabolites of interest with average birth weight of twin pairs across BTS (Beijing), LoTiS (Chongqing), and PETS (Melbourne) cohorts, analyzed using a generalized estimating equation (GEE) modelling. The red, blue, and green lines represent the 95% confidence intervals (CI) for the association between umbilical cord plasma metabolites and average birth weight in BTS, LoTiS, and PETS groups, respectively. Metabolites to the right of zero point (vertical dashed line) are positively correlated with average birth weight, whereas metabolites to the left are negatively correlated. Filled symbols indicate *p*-values less than 0.05, while unfilled denote non-significant correlations. **(B)** The linear regression between average neonatal birth weight and significant metabolite concentrations for between twin pairs in Beijing (purple lines), Chongqing (green lines), and Melbourne (yellow lines). The shade around the linear regression trendline displays the 95% CI.

Of the 33 total metabolites found to be variable across cohorts (Figure 5), 11,14-eicosadienoic showed a significant positive association with the average birth weight in all three groups by GEE analysis, whereas 3-hydroxyoctanoic acid was positively associated with the average birth weight in both Chinese twin groups and histidine was positively associated with the average birth weight in LoTiS and PETS twins (Figure 5; Supplementary Figure S4). We found caffeine, hexanoic acid, and glycine were to be significantly positively correlated with the average birth weight in the BTS, while gamma-linolenic acid, linoleic acid, decanoic acid and octanoic acid were positively correlated with the average birth weight in LoTiS (Figure 5; Supplementary Figure S4). Tryptophan was significantly positively correlated with the average birth weight in PETS, while 2-aminobutyric was significantly negatively correlated with the average birth weight in PETS. 4-methyl-2-oxopentanoic acid and 3-methyl-2-oxopentanoic acid were significantly negatively correlated with the average birth weight in PETS (Figure 5; Supplementary Figure S4).

### 3.5 Pathway analysis of specific metabolites associated with birth weight discordance

**Figure 6:**
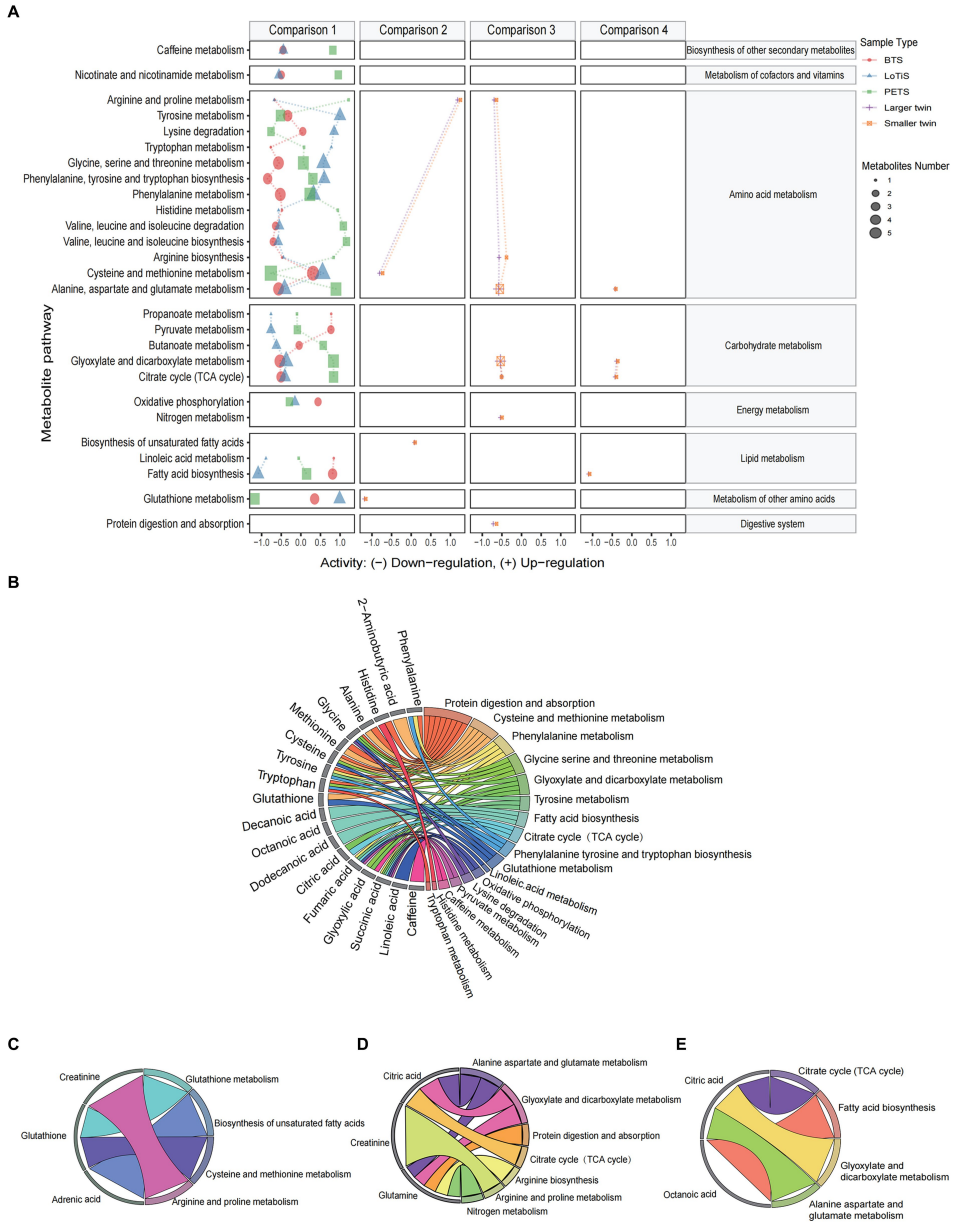
The metabolic pathway enrichment of the umbilical cord plasma from MCDA twins. **(A)** Predicated metabolic pathway activities for comparisons 1–4 using log2 (fold change). The plots on the right side of 0 represent upregulated metabolic activity, whereas the plots on the left side of 0 represent downregulated metabolic activity. The size of the dot is positively correlated with the enrichment ratio of the pathway computed by metabolite hits. Only the significant metabolites with *q*-values less than 0.05 are plotted. The indications of colour are as follows: BTS (red), LoTiS (blue), PETS (green), larger twin (purple), and small twin (orange). **(B–E)** Chord plots show the inter-relationship between metabolites and metabolic pathways. The four chord plots display the role of specific metabolites of interest from comparisons 1–4 (*p* < 0.05) to participate in different significant metabolic pathways.

Based on changes in cord blood metabolites, we annotated metabolic pathways using the KEGG metabolic framework. The analysis of predicted metabolic pathway activities for comparisons 1–4 is shown in Figure 6A. Most differential metabolic pathways were found in comparison 1. Particularly, caffeine metabolism, nicotinate and nicotinamide metabolism, valine leucine and isoleucine metabolism, alanine aspartate and glutamate metabolism, glyoxylate and dicarboxylate metabolism, and TCA cycle were downregulated in LoTiS and BTS, whereas these pathways were upregulated in PETS. And we found tyrosine metabolism, lysine degradation, glycine serine and threonine metabolism, phenylalanine tyrosine and tryptophan metabolism, and glutathione metabolism were higher metabolic activity in LoTiS relative to both BTS and PETS. Similar metabolic changes were observed within larger and smaller co-twin pairs in comparisons 2–4. In addition, chord plots were used to link the differentiated metabolites into significant metabolic pathways in each of the four comparisons (Figures 6B–E).

## 4 Discussion

In this study, we compared the umbilical cord blood plasma metabolome of MCDA twins derived from different regional, ethnic, and cultural contexts and explored the link of specific metabolites to birth weight. Umbilical cord plasma from the largely European PETS twins (Melbourne, Australia) exhibited profound disparity compared to those of Chinese origin (LoTiS and BTS). This was associated with significantly higher levels of caffeine, saturated fatty acids, and phenylalanine in PETS. On the other hand, several metabolites, such as glutathione, gamma-linolenic acid, and tyrosine, were highest in LoTiS (relative to PETS or BTS), while succinic acid and tryptophan were highest and lowest in BTS, respectively. It is highly likely that these metabolic discrepancies in blood plasma at birth are influenced by a range of factors including genetic and ethnic differences, dietary, and lifestyle factors.

### 4.1 Higher caffeine levels in PETS newborn plasma may derive from both intrinsic and dietary/lifestyle factors

Caffeine readily crosses the placental barrier, exposing the fetus to a concentration similar to maternal systemic levels (26, 27). In adults, caffeine is metabolized in the liver to paraxanthine, theobromine, and theophylline by cytochrome P450 enzymes. However, this system is not fully developed until infancy, so the clearance of fetal caffeine is fully dependent on maternal metabolism (28, 29).

The half-life of caffeine within the maternal circulation is extended from 5 h to about 18 h by the third trimester of pregnancy, which could rise to three times higher caffeine levels relative to non-pregnant women by the end of pregnancy (30, 31). However, this is unlikely to be relevant here, where no significant differences in gestational age were reported across twin cohorts. The higher level of plasma caffeine in PETS MCDA twins compared with LoTiS and BTS (Figure 4A) and the associated downregulation of caffeine metabolism in Chinese twins (LoTiS and BTS) compared to PETS (Figure 6) are likely due to a combination of previously reported genetic variation in enzymes (32, 33) associated with caffeine metabolism in addition to well-known differences in lifestyles/diets. Denden et al. (33) reported that genetics and ethnicity also influence caffeine metabolism. They found a significant association between genotype CYP1A2 rs762551 AA and higher coffee intake, especially in males, younger age groups, and individuals of Caucasian ethnicity, but not found in female, older, and Asian populations. Caffeine is most commonly consumed via coffee as well as foods including chocolate, cocoa foods, and some beverages (e.g., tea, soft drinks, and energy drinks) (34). Indeed, caffeine consumption in Australia is predominantly acquired from coffee, while in China is mainly contributed by green tea (35). Despite both espresso coffee and oolong tea containing between 30 and 100 mg of caffeine, the lower caffeine intake in China perhaps may relate to the Chinese advising pregnant and lactating women to minimize their intake of caffeine or caffeine-containing beverages (35). In addition, there are controversial reports regarding the relationship between maternal caffeine intake during gestation and perinatal outcomes. Brent et al. (36) found that moderate to high caffeine intake during pregnancy did not appear to elevate the risk of congenital malformations, miscarriage, or growth restriction. Other studies, however, concluded that both high intake (≥350 mg/day) and moderate intake (150 to 349 mg/day) by pregnant women were associated with a higher risk of delivering infants with low birth weight (37, 38) and pregnancy loss (39). The American College of Obstetricians and Gynecologists recommended that pregnant women limit caffeine consumption to less than 200 mg per day in 2010 (40). Several studies have reported that caffeine may disrupt fetal growth by blocking adenosine receptors and inhibiting cyclic nucleotide phosphodiesterase (PDE) (41). Caffeine acts as an adenosine receptor antagonist, leading to acute maternal hypoxia (42) and adversely impacting fetal cardiovascular and growth (43). In addition, when PDE is suppressed by caffeine, the levels of cyclic adenosine monophosphate (cAMP) will be elevated because PDE degrades cAMP, which may compromise fetal growth (44). Similarly, our study found that the Melbourne twins’ birth weight and birth weight discordance were lower and greater than the Chinese twins’ respectively. Reduced average birth weight and greater birth weight discordance between larger and smaller co-twins increase the risk of preterm birth (45), intrauterine growth restriction (46), and infant cognitive development retardation (47, 48). Thus, moderately reduced caffeine intake could be recommended for pregnant women.

### 4.2 Elevated saturated fatty acid levels and phenylalanine levels in PETS may be associated with maternal obesity

Pregnant women in Melbourne exhibited a significantly higher maternal BMI (Table 1), while their newborn twins showed significantly higher levels of plasma saturated fatty acids, such as hexanoic acid, dodecanoic acid, and pentadecanoic acid compared to those of Chinese ethnicity (LoTiS and BTS) (Figure 4). These saturated fatty acids are predominantly acquired from animal products (butter, lard) and tropical plant oils (e.g., coconut, palm) (49). Australians have one of the highest proportions of saturated fats intake (13.6%) in the world, which is almost two-fold higher than East Asian (7.4%) (50). DiNicolantonio et al. (51) demonstrated that long-chain saturated fatty acids including stearic acid (large quantities in butter) and palmitic acid (found in palm oil) are more likely to promote insulin resistance, inflammation, and fat storage. Another study on the association between maternal and fetal erythrocyte fatty acid profiles reported that pentadecanoic acids (C15:0) were detected at higher concentrations in smaller for gestational age (SGA) infants (52). The same study also indicated that both maternal and fetal saturated fatty acids are negatively associated with birth weight (52). Furthermore, phenylalanine also showed a significantly elevated level in PETS women than in Chinese cities (LoTiS and BTS). Phenylalanine is rich in dairy products and meat, including eggs, milk, and beef. Indeed, Oberbach et al. (53) demonstrated that obese individuals have higher blood phenylalanine levels. Libert et al. (54) also noted that plasma phenylalanine levels were associated with obesity, metabolic dysregulation, and diabetic pathophysiology (*p* < 0.0001). The authors suggested that this observation might be related to insulin resistance, the generation of reactive oxygen species, or early end-organ dysfunction. Furthermore, one hypothesis suggested that elevated phenylalanine levels in plasma may be related to liver dysfunction associated with metabolic unwellness, resulting in decreased phenylalanine and tyrosine metabolism (54–56). Therefore, we propose that the high levels of saturated fatty acids and phenylalanine in newborn plasma may reflect the higher BMI of mothers during pregnancy. Further research should be performed to validate our observation.

### 4.3 Differences between Chinese twins of different geographic locations: the impact of diet and pollution levels

Northern and southern China have divergent dietary cultures. The main staple food of Northern Chinese is predominantly processed carbohydrates, particularly noodles. In contrast, people in Chongqing, located in southwest China, tend to eat more spicy foods and animal offal. Spicy hotpot is a representative of Chongqing’s food culture. A cross-sectional survey of obesity among Chinese adults found that the rate of obesity among women in Beijing (northern China) was higher than that of women in Chongqing (57). Indeed, the LoTiS displayed the lowest maternal BMI, smallest twin birth weight discordance in this study, and highest normal newborn birth weight (Table 1). We have also found several metabolite signatures, including glutathione, gamma-linolenic acid, and tyrosine, that seem to reflect the eating culture of Chongqing. Particularly, glutathione exhibited elevated concentrations in the umbilical blood and showed a positive correlation with birth weight discordance in the LoTiS cohort (Figures 2D,E). *Zanthoxylum bungeanum maxim*. (ZBM) and *Capsicum annuum L*. (CAL) are common food additives in the Chongqing hotpot. Current research has reported that capsaicin in CAL, flavonoids, sanshool, and phenols in ZBM showed strong antioxidant effects (58, 59). Antioxidant supplementation has been reported to exhibit positive effects in various pathways such as strengthening blood circulation in the endometrium, reducing hyperandrogenism, reducing insulin resistance, and having positive effects on prostaglandin synthesis and steroid formation (60–63). Supplement of capsaicin in obese rats can relieve the hepatic oxidative stress induced by a high-fat diet (64). Capsaicin was also found to protect enzymes and proteins from radiation-induced oxidative damages, including the important endogenous antioxidant glutathione (GSH) (65). Studies have shown the beneficial effects of perinatal diet/injected antioxidants on pregnancy outcomes and growth performance of suckling kids of goats (66, 67). Many studies have reported the synergistic antioxidant effect of ZBM and CAL (68, 69). For instance, ZBM and CAL showed a remarkable antioxidant synergistic index (SI = 1.33) at 3:1 of ZBM/CAL ratio (70). Furthermore, tyrosine is the amino acid that constitutes protein, animal offal, and beef. Gamma-linolenic acid is an omega-6 fatty acid that can be produced through linoleic acid accounting for 40%–60% of total fatty acids in sesame oil (71), which is a common additive in Chongqing hotpot to make the spicy taste of the food less prominent. Chongqing’s characteristic diet is therefore likely associated with higher levels of tyrosine (linked to various amino acid metabolism; Figure 6) and gamma-linolenic acid, each of which was associated with higher birth weight (Figure 5A). Thus, we speculate that antioxidant-rich diets in Chongqing women may facilitate higher infant growth than in Beijing and Melbourne twins.

Epidemiological studies have shown that exposure to ambient air pollution impacts an individual’s metabolism (72). We found that the BTS showed a higher level of succinic acid and a lower level of tryptophan in umbilical blood (Figure 4D). Furthermore, we also found a positive correlation between tryptophan and average fetal birth weight in BTS (Figure 5A). Moreover, the World Air Quality Report showed that China’s air quality has been poorer than Melbourne’s in recent years, with Beijing’s “dustpan-shaped” terrain combined with heating, motor vehicle and industrial emissions making its air pollution more severe than Chongqing’s (73). For instance, in the September 2018 China City Air Quality Report, Chongqing’s PM_2.5_ concentration was 26 μg/m3, and Beijing’s PM_2.5_ concentration was 50 μg/m3 (74). Especially, particle matters with an aerodynamic diameter of less than 2.5 μm (PM_2.5_) have a greater impact on the health of pregnant women (75). A study found that a 5 μg/m^3^ increase in ambient PM_2.5_ concentration during pregnancy was associated with an increased risk of low birth weight (<2,500 g) at term [adjusted odds ratio (OR) 1.18, 95% CI 1.06–1·33] (76) Consistently, a longitudinal follow-up of 73 healthy adults living in Beijing from 2014 to 2016 found that short-term exposure to ambient air pollution can alter the metabolic profile and decrease plasma levels of amino acids (77). Another study showed that middle-aged mice (10 months) exposed to 3 mg/kg PM_2.5_ for 4 weeks elevated succinic acid levels by accelerating the tricarboxylic acid (TCA) cycle to offset energy requirements (78).

The strengths of our study include the application of an untargeted metabolomic approach to three uniquely diverse populations of newborn twins. The analysis of three distinct populations offers insights into a range of factors that may impact the newborn metabolome, including intrinsic factors (ethnicity/genetics), lifestyle and environment. Future analysis may benefit from an increased sample size from each cohort and the inclusion of further population centers from which individuals can be drawn to increase the comprehensiveness of the study. The results presented here from machine learning approaches represent a training model and need to be independently validated/replicated in other cohorts and sets of umbilical blood of twin pregnancies. In future studies, it will be interesting to assess the impact of other factors (such as genetic variation, socioeconomic status, or maternal smoking) on differences in the metabolomic profile of MCDA twins from different populations at birth. Further, the inclusion of dizygotic twins (with variable genetic profiles), and twins of different ages, would enable a more direct assessment of the role of genetic factors in driving metabolic differences. Despite clear differences in metabolites between larger and smaller co-twin pairs according to location (e.g., antioxidants in Melbourne twins, itaconic acid in Beijing twins, and citric acid for Chinese twins), the specific factors leading to these distinct relationships require further investigation. Future studies should include direct assessments of diet and lifestyle factors, plus environmental measures (e.g., pollution).

## 5 Conclusion

**Figure 7:**
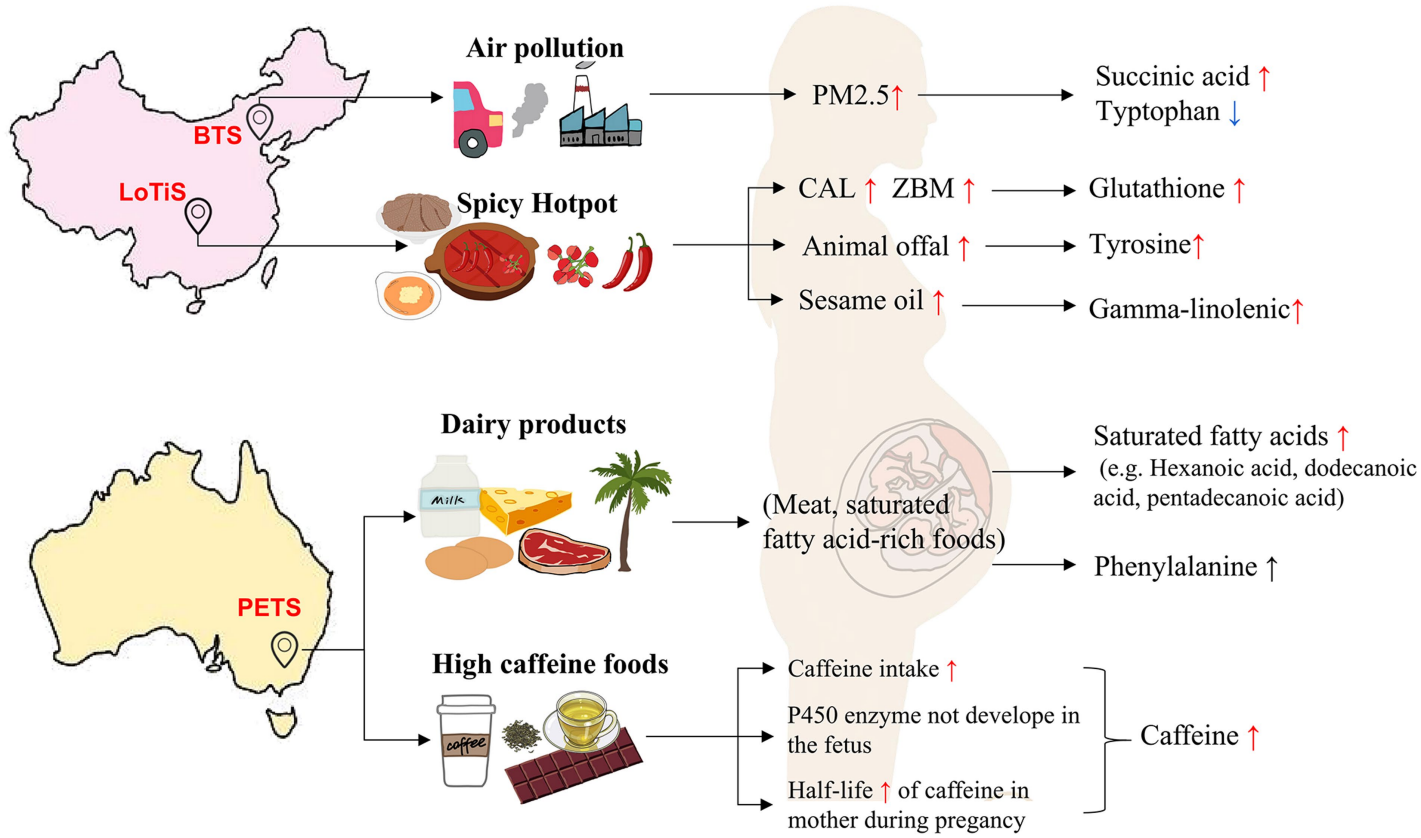
The influence of diet and environmental factors on the umbilical cord blood metabolome of MCDA twins between different regions. Air pollution may contribute to a higher and lower level of succinic acid and tryptophan in BTS twin, respectively. The spicy food culture in Chongqing may result in higher levels of glutathione, tyrosine, and gamma-linolenic in LoTiS twins’ cord blood. The most abundant caffeine levels in PETS twins could be associated with a higher coffee, coke, and chocolate intake. Furthermore, increased phenylalanine and some saturated fatty acid levels in Melbourne twins may likely be attributed by a higher intake of dairy products, meats, and tropical plant oils. PM_2.5_, particle matters with an aerodynamic diameter less than 2.5 μm; ZBM, *Zanthoxylum bungeanum* Maxim; and CAL, *Capsicum annuum* L.

Our research applied a metabolomic approach to analyze the umbilical cord blood MCDA twins from three different populations. Large disparities in the metabolome profile between largely European PETS twins and ethnically Chinese twins were observed (LoTiS and BTS). This was particularly apparent for caffeine and saturated fatty acid levels (hexanoic acid, dodecanoic acid, and pentadecanoic acid), both higher in Melbourne twins, while spicy diets and air pollution may be related to LoTiS and BTS-specific metabolite levels, respectively, (Figure 7). This study revealed that the LoTiS MCDA twins exhibited the lowest level of discordance, primarily attributable to a combination of a healthier diet and reduced pollution levels. Our finding suggested that the fetal cord blood metabolome is influenced by a range of intrinsic (genetic, ethnic) and extrauterine factors such as geographic regions, unique dietary habits, and environmental pollution. This study provides new insights into the regional effects of the fetal cord blood metabolome on perinatal growth and development.

## Data availability statement

The raw data supporting the conclusions of this article will be made available by the authors, without undue reservation.

## Ethics statement

The studies involving humans were approved by and carried out in accordance with the principles set out in the Declaration of Helsinki and approved by the Ethical Committee of Chongqing Medical University (201530), and the Ethical Committee of Peking University Third Hospital. PETS was carried out with appropriate human ethics approvals from the Royal Women’s Hospital (06/21), Mercy Hospital for Women (R06/30), and Monash Medical Centre (06117C), Melbourne. The studies were conducted in accordance with the local legislation and institutional requirements. Written informed consent for participation in this study was provided by the participants’ legal guardians/next of kin.

## Author contributions

HJ: Writing – original draft. T-LH: Conceptualization, Writing – review & editing, Writing – original draft. JY: Conceptualization, Writing – review & editing. YY: Writing – review & editing, Data curation. FW: Writing – review & editing. YC: Writing – review & editing. NH: Writing – review & editing. TM: Writing – review & editing. JC: Writing – review & editing. KS: Writing – review & editing. BN: Writing – review & editing. PB: Writing – review & editing. HZ: Writing – review & editing. YW: Writing – review & editing. LW: Conceptualization, Writing – review & editing. RS: Writing – review & editing.

## Footnotes

^1^ http://www.omicshare.com/tools

## Supplementary material

Supplementary Figure S1 Principal component analysis (PCA) of the umbilical cord plasma between female and male twins for **(A)** PETS - Comparison 2, **(B)** BTS - Comparison 3, and **(C)** LoTiS - Comparison 4.

Supplementary Figure S2 Partial least squares discriminant analysis (PLS-DA) of the umbilical cord plasma metabolome among Melbourne, Beijing and Chongqing MCDA twins, including a measure of prediction model performance (right bar graphs). The right bar graphics are leave-one-out cross validations (LOOCV), where R2 indicates how well the model explains the data and Q2 indicates reproducibility of the PLS-DA model. **(A)** PETS - Comparison 2. **(B)** BTS - Comparison 3. **(C)** LoTiS - Comparison 4.

Supplementary Figure S3 Box plots show the specific metabolites except for those shown in Figure 4. Statistical significance between groups were determined using logistic regression (**p* <0.05; ***p* < 0.01; ****p* < 0.001).

Supplementary Figure S4 The linear regression between average neonatal birth weight and significant metabolite concentrations for between twin pairs in Beijing (purple lines), Chongqing (green lines), and Melbourne (yellow lines) except for those shown in Figure 5. The shade around the linear regression trendline displays the 95% CI.

## References

[1] Bianco-Miotto T Craig JM Gasser YP van Dijk SJ Ozanne SE. Epigenetics and DOHaD: from basics to birth and beyond. J Dev Orig Health Dis. (2017) 8:513–9. doi: 10.1017/S2040174417000733 28889823

[2] Melicher D Illés A Pállinger É Kovács ÁF Littvay L Tárnoki ÁD. Tight co-twin similarity of monozygotic twins for hTERT protein level of T cell subsets, for telomere length and mitochondrial DNA copy number, but not for telomerase activity. Cell Mol Life Sci. (2018) 75:2447–56. doi: 10.1007/s00018-017-2738-z, PMID: 29290038 PMC11105316

[3] Lewi L Jani J Blickstein I Huber A Gucciardo L Van Mieghem T. The outcome of monochorionic diamniotic twin gestations in the era of invasive fetal therapy: a prospective cohort study. Am J Obstet Gynecol. (2008) 199:514.e1–8. doi: 10.1016/j.ajog.2008.03.050, PMID: 18533114

[4] Fick AL Feldstein VA Norton ME Wassel Fyr C Caughey AB Machin GA. Unequal placental sharing and birth weight discordance in monochorionic diamniotic twins. Am J Obstet Gynecol. (2006) 195:178–83. doi: 10.1016/j.ajog.2006.01.015, PMID: 16643828

[5] Cosmi E Visentin S Favretto D Tucci M Ragazzi E Viel G. Selective intrauterine growth restriction in monochorionic twin pregnancies: markers of endothelial damage and metabolomic profile. Twin Res Hum Genet. (2013) 16:816–26. doi: 10.1017/thg.2013.33, PMID: 23701694

[6] Aquino C Rodrigues Baião AE de Carvalho PRN. Perinatal outcome of selective intrauterine growth restriction in monochorionic twins: evaluation of a retrospective cohort in a developing country. Twin Res Hum Genet. (2021) 24:37–41. doi: 10.1017/thg.2021.7 33745489

[7] Jelenkovic A Yokoyama Y Sund R Honda C Bogl LH Aaltonen S. Zygosity differences in height and body mass index of twins from infancy to old age: a study of the CODATwins project. Twin Res Hum Genet. (2015) 18:557–70. doi: 10.1017/thg.2015.57, PMID: 26337138 PMC4605819

[8] Yokoyama Y Jelenkovic A Sund R Sung J Hopper JL Ooki S. Twin’s birth-order differences in height and body mass index from birth to old age: a pooled study of 26 twin cohorts participating in the CODATwins project. Twin Res Hum Genet. (2016) 19:112–24. doi: 10.1017/thg.2016.11, PMID: 26996222 PMC5100672

[9] Martino D Loke YJ Gordon L Ollikainen M Cruickshank MN Saffery R. Longitudinal, genome-scale analysis of DNA methylation in twins from birth to 18 months of age reveals rapid epigenetic change in early life and pair-specific effects of discordance. Genome Biol. (2013) 14:R42. doi: 10.1186/gb-2013-14-5-r42, PMID: 23697701 PMC4054827

[10] Gordon L Joo JE Powell JE Ollikainen M Novakovic B Li X. Neonatal DNA methylation profile in human twins is specified by a complex interplay between intrauterine environmental and genetic factors, subject to tissue-specific influence. Genome Res. (2012) 22:1395–406. doi: 10.1101/gr.136598.111, PMID: 22800725 PMC3409253

[11] Yang J Wei Y Qi H Yin N Yang Y Li Z. Neonatal hair profiling reveals a metabolic phenotype of monochorionic twins with selective intrauterine growth restriction and abnormal umbilical artery flow. Mol Med. (2020) 26:37. doi: 10.1186/s10020-020-00160-8, PMID: 32357834 PMC7193362

[12] Yang J Hou L Wang J Xiao L Zhang J Yin N. Unfavourable intrauterine environment contributes to abnormal gut microbiome and metabolome in twins. Gut. (2022) 71:2451–62. doi: 10.1136/gutjnl-2021-326482, PMID: 35387876 PMC9664093

[13] Tong C Wen L Xia Y Leong P Wang L Fan X. Protocol for a longitudinal twin birth cohort study to unravel the complex interplay between early-life environmental and genetic risk factors in health and disease: the Chongqing longitudinal twin study (LoTiS). BMJ Open. (2018) 8:e017889. doi: 10.1136/bmjopen-2017-017889, PMID: 29472256 PMC5855313

[14] Tong C Wen L Wang L Fan X Zhao Y Liu Y. Cohort profile: the Chongqing longitudinal twin study (LoTiS). Int J Epidemiol. (2022) 51:e256–66. doi: 10.1093/ije/dyab264 35051283

[15] Wang L Han TL Luo X Li S Young T Chen C. Metabolic biomarkers of monochorionic twins complicated with selective intrauterine growth restriction in cord plasma and placental tissue. Sci Rep. (2018) 8:15914. doi: 10.1038/s41598-018-33788-y, PMID: 30374111 PMC6206027

[16] Dias DA Koal T. Progress in metabolomics standardisation and its significance in future clinical laboratory medicine. EJIFCC. (2016) 27:331–43. PMID: 28149265 PMC5282916

[17] Beale DJ Jones OA Karpe AV Dayalan S Oh DY Kouremenos KA. A review of analytical techniques and their application in disease diagnosis in breathomics and salivaomics research. Int J Mol Sci. (2016) 18:24. doi: 10.3390/ijms18010024, PMID: 28025547 PMC5297659

[18] Nassar AF Wu T Nassar SF Wisnewski AV. UPLC-MS for metabolomics: a giant step forward in support of pharmaceutical research. Drug Discov Today. (2017) 22:463–70. doi: 10.1016/j.drudis.2016.11.020, PMID: 27919805 PMC5721520

[19] Saffery R Morley R Carlin JB Joo JH Ollikainen M Novakovic B. Cohort profile: the peri/post-natal epigenetic twins study. Int J Epidemiol. (2012) 41:55–61. doi: 10.1093/ije/dyr140, PMID: 22422448

[20] Smart KF Aggio RB Van Houtte JR Villas-Bôas SG. Analytical platform for metabolome analysis of microbial cells using methyl chloroformate derivatization followed by gas chromatography-mass spectrometry. Nat Protoc. (2010) 5:1709–29. doi: 10.1038/nprot.2010.108, PMID: 20885382

[21] Han TL Cannon RD Gallo SM Villas-Boas SG. A metabolomic study of the effect of *Candida albicans* glutamate dehydrogenase deletion on growth and morphogenesis. npj Biofilms Microbiomes. (2019) 5:13. doi: 10.1038/s41522-019-0086-5, PMID: 30992998 PMC6453907

[22] Behrends V Tredwell GD Bundy JG. A software complement to AMDIS for processing GC-MS metabolomic data. Anal Biochem. (2011) 415:206–8. doi: 10.1016/j.ab.2011.04.009, PMID: 21575589

[23] Guo G McKenzie Elizabeth J. Jones B Zarate E Seymour J Baker PN. MassOmics: an R package of a cross-platform data processing pipeline for large-scale GC-MS untargeted metabolomics datasets. (2021). Available at: https://zenodo.org/records/4961895.

[24] Xia J Sinelnikov IV Han B Wishart DS. MetaboAnalyst 3.0—making metabolomics more meaningful. Nucleic Acids Res. (2015) 43:W251–7. doi: 10.1093/nar/gkv380, PMID: 25897128 PMC4489235

[25] Walter W Sánchez-Cabo F Ricote M. GOplot: an R package for visually combining expression data with functional analysis. Bioinformatics. (2015) 31:2912–4. doi: 10.1093/bioinformatics/btv300 25964631

[26] James JE. Maternal caffeine consumption and pregnancy outcomes: a narrative review with implications for advice to mothers and mothers-to-be. BMJ Evid Based Med. (2021) 26:114–5. doi: 10.1136/bmjebm-2020-111432 PMC8165152 32843532

[27] Darakjian LI Kaddoumi A. Physiologically based pharmacokinetic/pharmacodynamic model for caffeine disposition in pregnancy. Mol Pharm. (2019) 16:1340–9. doi: 10.1021/acs.molpharmaceut.8b01276, PMID: 30689395

[28] Pearlman SA Duran C Wood MA Maisels MJ Berlin CM Jr. Caffeine pharmacokinetics in preterm infants older than 2 weeks. Dev Pharmacol Ther. (1989) 12:65–9. doi: 10.1159/000480966, PMID: 2714159

[29] Klebanoff MA Levine RJ Clemens JD Wilkins DG. Maternal serum caffeine metabolites and small-for-gestational age birth. Am J Epidemiol. (2002) 155:32–7. doi: 10.1093/aje/155.1.32, PMID: 11772782

[30] Liang L Rasmussen MH Piening B Shen X Chen S Röst H. Metabolic dynamics and prediction of gestational age and time to delivery in pregnant women. Cells. (2020) 181:1680–92.e15. doi: 10.1016/j.cell.2020.05.002, PMID: 32589958 PMC7327522

[31] Knutti R Rothweiler H Schlatter C. Effect of pregnancy on the pharmacokinetics of caffeine. Eur J Clin Pharmacol. (1981) 21:121–6. doi: 10.1007/BF00637512 7341280

[32] Nehlig A. Interindividual differences in caffeine metabolism and factors driving caffeine consumption. Pharmacol Rev. (2018) 70:384–411. doi: 10.1124/pr.117.014407 29514871

[33] Denden S Bouden B Haj Khelil A Ben Chibani J Hamdaoui MH. Gender and ethnicity modify the association between the CYP1A2 rs 762551 polymorphism and habitual coffee intake: evidence from a meta-analysis. Genet Mol Res. (2016) 15. doi: 10.4238/gmr.15027487, PMID: 27173183

[34] Abalo R. Coffee and caffeine consumption for human health. Nutrients. (2021) 13:2918. doi: 10.3390/nu13092918, PMID: 34578795 PMC8468147

[35] Reyes CM Cornelis MC. Caffeine in the diet: country-level consumption and guidelines. Nutrients. (2018) 10:1772. doi: 10.3390/nu10111772, PMID: 30445721 PMC6266969

[36] Brent RL Christian MS Diener RM. Evaluation of the reproductive and developmental risks of caffeine. Birth Defects Res B Dev Reprod Toxicol. (2011) 92:152–87. doi: 10.1002/bdrb.20288, PMID: 21370398 PMC3121964

[37] Chen LW Wu Y Neelakantan N Chong MF Pan A van Dam RM. Maternal caffeine intake during pregnancy is associated with risk of low birth weight: a systematic review and dose-response meta-analysis. BMC Med. (2014) 12:174. doi: 10.1186/s12916-014-0174-6, PMID: 25238871 PMC4198801

[38] Gleason JL Tekola-Ayele F Sundaram R Hinkle SN Vafai Y Buck Louis GM. Association between maternal caffeine consumption and metabolism and neonatal anthropometry: a secondary analysis of the NICHD fetal growth studies-singletons. JAMA Netw Open. (2021) 4:e213238. doi: 10.1001/jamanetworkopen.2021.3238, PMID: 33764424 PMC7994948

[39] Chen LW Wu Y Neelakantan N Chong MF Pan A van Dam RM. Maternal caffeine intake during pregnancy and risk of pregnancy loss: a categorical and dose-response meta-analysis of prospective studies. Public Health Nutr. (2016) 19:1233–44. doi: 10.1017/S1368980015002463, PMID: 26329421 PMC10271029

[40] ACOG CommitteeOpinion No. 462: Moderate caffeine consumption during pregnancy. Obstet Gynecol. (2010) 116:467–8. doi: 10.1097/AOG.0b013e3181eeb2a1 20664420

[41] Daly JW. Alkylxanthines as research tools. J Auton Nerv Syst. (2000) 81:44–52. doi: 10.1016/S0165-1838(00)00110-7, PMID: 10869699

[42] Fortier I Marcoux S Beaulac-Baillargeon L. Relation of caffeine intake during pregnancy to intrauterine growth retardation and preterm birth. Am J Epidemiol. (1993) 137:931–40. doi: 10.1093/oxfordjournals.aje.a116763, PMID: 8317450

[43] Momoi N Tinney JP Keller BB Tobita K. Maternal hypoxia and caffeine exposure depress fetal cardiovascular function during primary organogenesis. J Obstet Gynaecol Res. (2012) 38:1343–51. doi: 10.1111/j.1447-0756.2012.01880.x, PMID: 22612345 PMC3427416

[44] Bistoletti P Fredholm BB Lagercrantz H. Cyclic 3′,5′-adenosine monophosphate in umbilical cord blood of the newborn infant: relation to fetal stress and plasma catecholamines. Biol Neonate. (1981) 40:145–9. doi: 10.1159/000241483 6269661

[45] Cooperstock MS Tummaru R Bakewell J Schramm W. Twin birth weight discordance and risk of preterm birth. Am J Obstet Gynecol. (2000) 183:63–7. doi: 10.1016/S0002-9378(00)55604-X 10920310

[46] Swamy RS McConachie H Ng J Rankin J Korada M Sturgiss S. Cognitive outcome in childhood of birth weight discordant monochorionic twins: the long-term effects of fetal growth restriction. Arch Dis Child Fetal Neonatal Ed. (2018) 103:F512–6. doi: 10.1136/archdischild-2017-313691, PMID: 29500316

[47] Groene SG Tollenaar LSA Oepkes D Lopriore E van Klink JMM. The impact of selective fetal growth restriction or birth weight discordance on long-term neurodevelopment in monochorionic twins: a systematic literature review. J Clin Med. (2019) 8:944. doi: 10.3390/jcm8070944, PMID: 31261823 PMC6678939

[48] Halling C Malone FD Breathnach FM Stewart MC McAuliffe FM Morrison JJ. Neuro-developmental outcome of a large cohort of growth discordant twins. Eur J Pediatr. (2016) 175:381–9. doi: 10.1007/s00431-015-2648-8, PMID: 26490567

[49] Tvrzicka E Kremmyda LS Stankova B Zak A. Fatty acids as biocompounds: their role in human metabolism, health and disease--a review. Part 1: classification, dietary sources and biological functions. Biomed Pap Med Fac Univ Palacky Olomouc Czech Repub. (2011) 155:117–30. doi: 10.5507/bp.2011.038, PMID: 21804620

[50] Micha R Khatibzadeh S Shi P Fahimi S Lim S Andrews KG. Global, regional, and national consumption levels of dietary fats and oils in 1990 and 2010: a systematic analysis including 266 country-specific nutrition surveys. BMJ. (2014) 348:2272. doi: 10.1136/bmj.g2272, PMID: 24736206 PMC3987052

[51] DiNicolantonio JJ O’Keefe JH. Good fats versus bad fats: a comparison of fatty acids in the promotion of insulin resistance, inflammation, and obesity. Mo Med. (2017) 114:303–7. 30228616 PMC6140086

[52] Cinelli G Fabrizi M Ravà L Signore F Vernocchi P Semeraro M. Association between maternal and Foetal erythrocyte fatty acid profiles and birth weight. Nutrients. (2018) 10:402. doi: 10.3390/nu10040402, PMID: 29570689 PMC5946187

[53] Oberbach A Blüher M Wirth H Till H Kovacs P Kullnick Y. Combined proteomic and metabolomic profiling of serum reveals association of the complement system with obesity and identifies novel markers of body fat mass changes. J Proteome Res. (2011) 10:4769–88. doi: 10.1021/pr2005555 21823675

[54] Libert DM Nowacki AS Natowicz MR. Metabolomic analysis of obesity, metabolic syndrome, and type 2 diabetes: amino acid and acylcarnitine levels change along a spectrum of metabolic wellness. PeerJ. (2018) 6:e5410. doi: 10.7717/peerj.5410 30186675 PMC6120443

[55] Fernstrom JD. Branched-chain amino acids and brain function. J Nutr. (2005) 135:1539s–46s. doi: 10.1093/jn/135.6.1539S 15930466

[56] Newgard CB An J Bain JR Muehlbauer MJ Stevens RD Lien LF. A branched-chain amino acid-related metabolic signature that differentiates obese and lean humans and contributes to insulin resistance. Cell Metab. (2009) 9:311–26. doi: 10.1016/j.cmet.2009.02.002, PMID: 19356713 PMC3640280

[57] Zhang X Zhang M Zhao Z Huang Z Deng Q Li Y. Geographic variation in prevalence of adult obesity in China: results from the 2013–2014 national chronic disease and risk factor surveillance. Ann Intern Med. (2020) 172:291–3. doi: 10.7326/M19-0477 31658469

[58] Perucka I Materska M. Phenylalanine ammonia-lyase and antioxidant activities of lipophilic fraction of fresh pepper fruits *Capsicum annum* L. Innov Food Sci Emerg Technol. (2001) 2:189–92. doi: 10.1016/S1466-8564(01)00022-4

[59] Deng S Rong H Tu H Zheng B Mu X Zhu L. Molecular basis of neurophysiological and antioxidant roles of Szechuan pepper. Biomed Pharmacother. (2019) 112:108696. doi: 10.1016/j.biopha.2019.108696, PMID: 30818139

[60] Hussain T Murtaza G Metwally E Kalhoro DH Kalhoro MS Rahu BA. The role of oxidative stress and antioxidant balance in pregnancy. Mediat Inflamm. (2021) 2021:1–11. doi: 10.1155/2021/9962860 PMC8490076 34616234

[61] Thomson RL Spedding S Buckley JD. Vitamin D in the aetiology and management of polycystic ovary syndrome. Clin Endocrinol. (2012) 77:343–50. doi: 10.1111/j.1365-2265.2012.04434.x 22574874

[62] Lédée-Bataille N Olivennes F Lefaix JL Chaouat G Frydman R Delanian S. Combined treatment by pentoxifylline and tocopherol for recipient women with a thin endometrium enrolled in an oocyte donation programme. Hum Reprod. (2002) 17:1249–53. doi: 10.1093/humrep/17.5.1249 11980747

[63] Mirończuk-Chodakowska I Witkowska AM Zujko ME. Endogenous non-enzymatic antioxidants in the human body. Adv Med Sci. (2018) 63:68–78. doi: 10.1016/j.advms.2017.05.005 28822266

[64] Tanrıkulu-Küçük S Başaran-Küçükgergin C Seyithanoğlu M Doğru-Abbasoğlu S Koçak H Beyhan-Özdaş Ş. Effect of dietary curcumin and capsaicin on testicular and hepatic oxidant-antioxidant status in rats fed a high-fat diet. Appl Physiol Nutr Metab. (2019) 44:774–82. doi: 10.1139/apnm-2018-0622 30605349

[65] Manjunatha H Srinivasan K. Hypolipidemic and antioxidant effects of curcumin and capsaicin in high-fat-fed rats. Can J Physiol Pharmacol. (2007) 85:588–96. doi: 10.1139/Y07-044, PMID: 17823620

[66] Afzal A Hussain T Hameed A. *Moringa oleifera* supplementation improves antioxidant status and biochemical indices by attenuating early pregnancy stress in Beetal goats. Front Nutr. (2021) 8:700957. doi: 10.3389/fnut.2021.700957, PMID: 34368210 PMC8342799

[67] Mahmood N Hameed A Hussain T. Vitamin E and selenium treatment alleviates saline environment-induced oxidative stress through enhanced antioxidants and growth performance in suckling kids of Beetal goats. Oxidative Med Cell Longev. (2020) 2020:1–16. doi: 10.1155/2020/4960507 PMC7563068 33082909

[68] Mao S Wang K Lei Y Yao S Lu B Huang W. Antioxidant synergistic effects of *Osmanthus fragrans* flowers with green tea and their major contributed antioxidant compounds. Sci Rep. (2017) 7:46501. doi: 10.1038/srep46501, PMID: 28422181 PMC5395974

[69] Jain DP Pancholi SS Patel R. Synergistic antioxidant activity of green tea with some herbs. J Adv Pharm Technol Res. (2011) 2:177–83. doi: 10.4103/2231-4040.85538, PMID: 22171315 PMC3217702

[70] Peng Q Lu Y Mo R He Q. Antioxidant and nitrite-scavenging activities of *Zanthoxylum bungeanum* maxim. And *Capsicum annuum* L.: a synergistic, additive or antagonistic effect of the extracts? Eur Food Res Technol. (2021) 247:2877–85. doi: 10.1007/s00217-021-03845-4

[71] Ramsden CE Zamora D Faurot KR MacIntosh B Horowitz M Keyes GS. Dietary alteration of n-3 and n-6 fatty acids for headache reduction in adults with migraine: randomized controlled trial. BMJ. (2021) 374:n1448. doi: 10.1136/bmj.n1448 34526307 PMC8244542

[72] Eze IC Schaffner E Foraster M Imboden M von Eckardstein A Gerbase MW. Long-term exposure to ambient air pollution and metabolic syndrome in adults. PLoS One. (2015) 10:e0130337. doi: 10.1371/journal.pone.0130337, PMID: 26103580 PMC4478007

[73] Zhang YL Cao F. Fine particulate matter (PM_2.5_) in China at a city level. Sci Rep. (2015) 5:14884. doi: 10.1038/srep14884, PMID: 26469995 PMC4606739

[74] Ministry of Ecology and Environment of People’s Republic of China. (2018) China City air quality report 2018-09-05 Available at: https://www.mee.gov.cn/hjzl/dqhj/cskqzlzkyb/201809/P020180905326235405574.pdf.

[75] Cohen AJ Brauer M Burnett R Anderson HR Frostad J Estep K. Estimates and 25-year trends of the global burden of disease attributable to ambient air pollution: an analysis of data from the global burden of diseases study 2015. Lancet. (2017) 389:1907–18. doi: 10.1016/S0140-6736(17)30505-6 28408086 PMC5439030

[76] Pedersen M Giorgis-Allemand L Bernard C Aguilera I Andersen AM Ballester F. Ambient air pollution and low birthweight: a European cohort study (ESCAPE). Lancet Respir Med. (2013) 1:695–704. doi: 10.1016/S2213-2600(13)70192-9 24429273

[77] Feng B Liu C Yi T Song X Wang Y Liu S. Perturbation of amino acid metabolism mediates air pollution associated vascular dysfunction in healthy adults. Environ Res. (2021) 201:111512. doi: 10.1016/j.envres.2021.111512 34166659

[78] Zhang Y Ji X Ku T Li B Li G Sang N. Ambient fine particulate matter exposure induces cardiac functional injury and metabolite alterations in middle-aged female mice. Environ Pollut. (2019) 248:121–32. doi: 10.1016/j.envpol.2019.01.080, PMID: 30784831

