# Supplementary figures and images for: Evidence for ethnicity and location as regulators of the newborn blood metabolome: a monozygous twin study
Source: Front Nutr. 2024 Jan 4;10:1259777. doi: 10.3389/fnut.2023.1259777 (PMC10794553; doi:10.3389/fnut.2023.1259777)

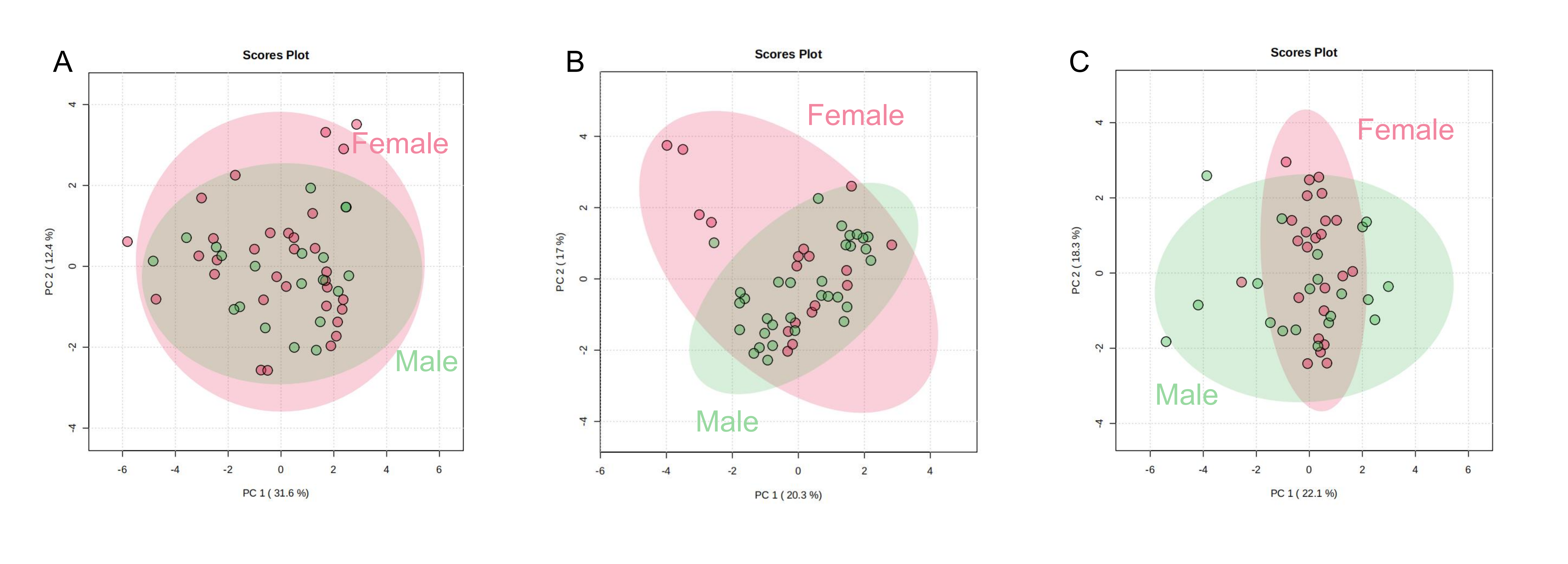

Supplement: Supplementary Figure S1 — Principal component analysis (PCA) of the umbilical cord plasma between female and male twins for (A) PETS - Comparison 2, (B) BTS - Comparison 3, and (C) LoTiS - Comparison 4. [file Image_1.PNG]

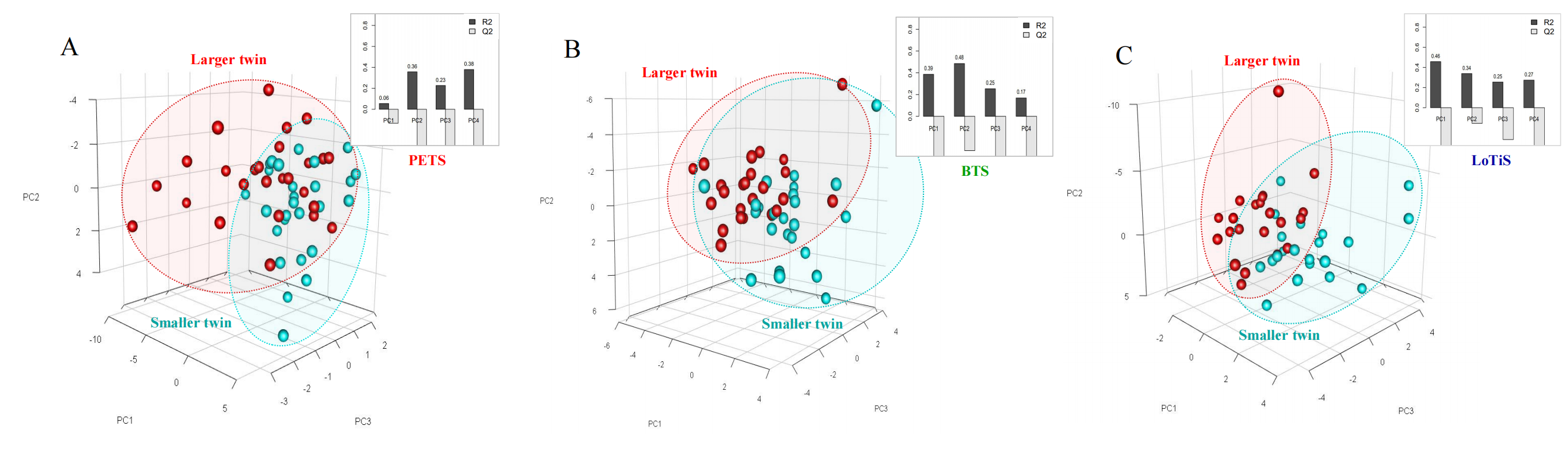

Supplement: Supplementary Figure S2 — Partial least squares discriminant analysis (PLS-DA) of the umbilical cord plasma metabolome among Melbourne, Beijing and Chongqing MCDA twins, including a measure of prediction model performance (right bar graphs). The right bar graphics are leave-one-out cross validations (LOOCV), where R2 indicates how well the model explains the data and Q2 indicates reproducibility of the PLS-DA model. (A) PETS - Comparison 2. (B) BTS - Comparison 3. (C) LoTiS - Comparison 4. [file Image_2.TIF]

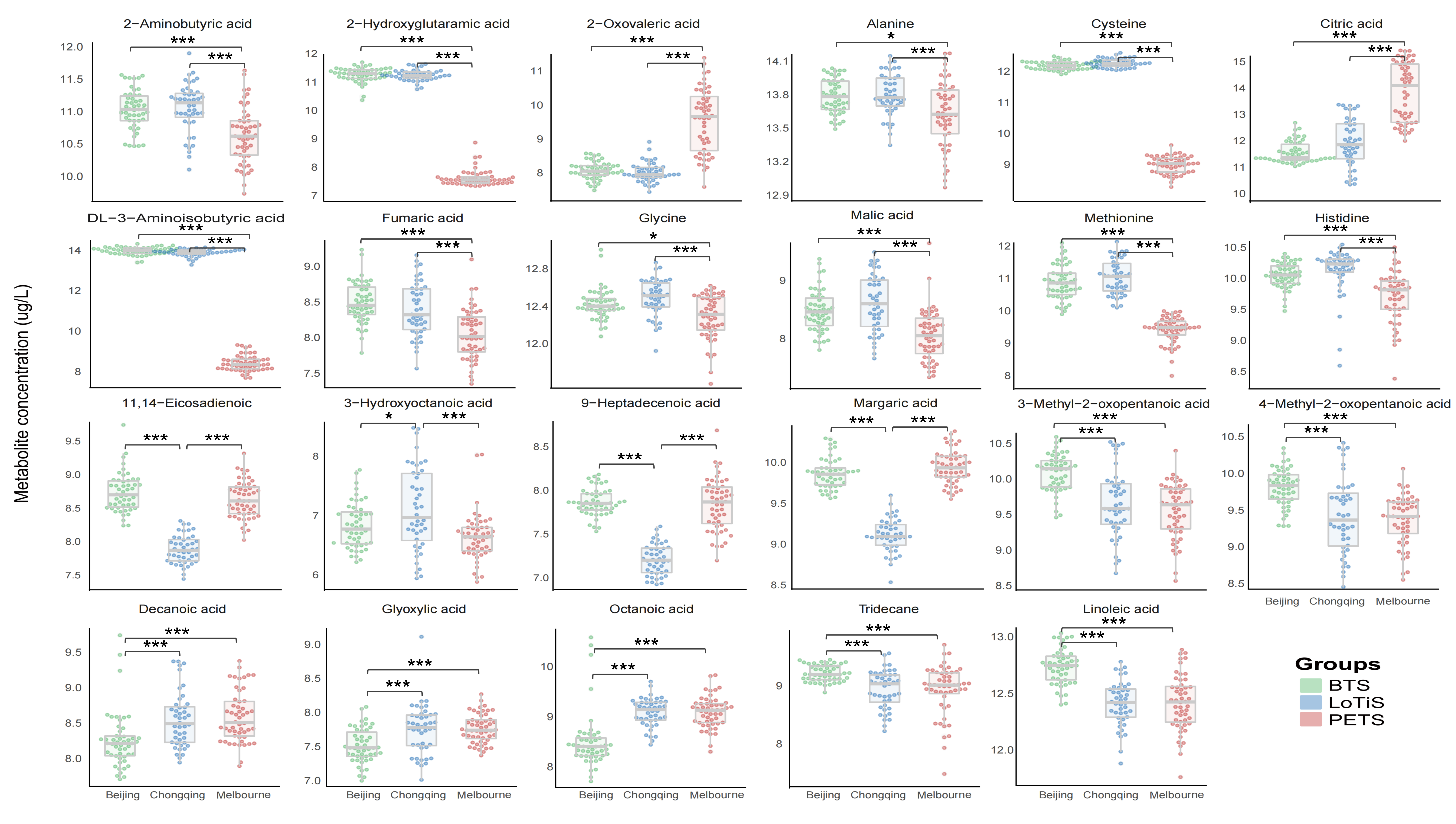

Supplement: Supplementary Figure S3 — Box plots show the specific metabolites except for those shown in Figure 4. Statistical significance between groups were determined using logistic regression (*p <0.05; **p < 0.01; ***p < 0.001). [file Image_3.PNG]

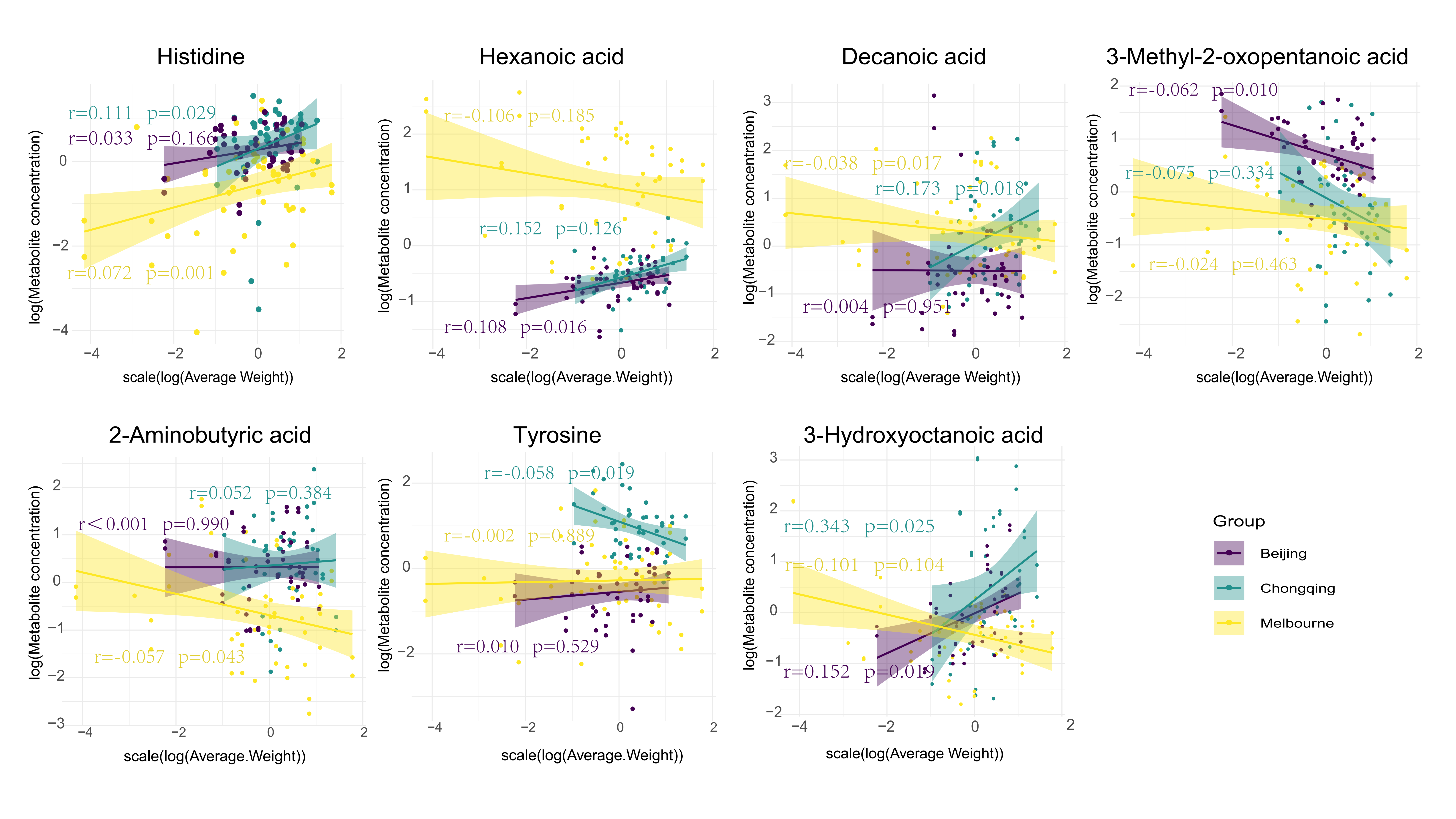

Supplement: Supplementary Figure S4 — The linear regression between average neonatal birth weight and significant metabolite concentrations for between twin pairs in Beijing (purple lines), Chongqing (green lines), and Melbourne (yellow lines) except for those shown in Figure 5. The shade around the linear regression trendline displays the 95% CI. [file Image_4.PNG]
